# Genetic characterization of Gaddi goat breed of Western Himalayas using microsatellite markers

**DOI:** 10.14202/vetworld.2015.527-531

**Published:** 2015-04-22

**Authors:** Gurdeep Singh, Yashpal Thakur, Amitoz Kour, Varun Sankhyan, Sanjeet Katoch

**Affiliations:** 1 Department of Animal Genetics and Breeding, CSK Himachal Pradesh Krishi Vishvavidyalaya, College of Veterinary and Animal Sciences, Holta, Palampur - 176 062, Himachal Pradesh, India; 2 Department of Veterinary Microbiology, CSK Himachal Pradesh Krishi Vishvavidyalaya, College of Veterinary and Animal Sciences, Holta, Palampur - 176 062, Himachal Pradesh, India

**Keywords:** characterization, Gaddi goat, microsatellite markers, Western Himalayas

## Abstract

**Aim:**

In the present study, genetic characterization of Gaddi goat breed, a native to north temperate western Himalayan region of India, was carried out for the purpose of breed characterization and assessing existing intra-population genetic diversity.

**Materials and Methods:**

Totally, 75 blood samples procured at random from genetically unrelated animals of two sexes and different age groups and true to breed type were collected from different locations in the breeding tract of these goats in Himachal Pradesh, of which only 51 samples with desired quantity and quality were subjected to further processing for DNA isolation. The multi-locus genotype data were generated on 51 Gaddi goats sampled across different regions of the breeding tract in Himachal Pradesh using 15 FAO recommended goat specific microsatellite markers, which gave amplification and observed and effective number of alleles, gene frequency, observed and expected heterozygosity were estimated through PopGene software (1.3.1).

**Results:**

A total of 135 distinct alleles were observed with mean observed and effective number of alleles as 9.0000±0.82 and 6.5874±0.56 respectively across all 15 studied loci. The maximum (15) alleles were contributed by loci DRBP1 and P19/DYA and the least (5) by SRCRSP5. The mean heterozygosity was observed to be 0.8347±0.01 ranging from 0.7584 (SRCRSP5) to 0.9156 (P19-DYA) across all loci. The mean observed (H_O_) and expected (H_E_) heterozygosities across all loci were 0.7484±0.02 and 0.8431±0.01 respectively. The polymorphism information content (PIC) value ranged from 0.7148 (SRCPS5) to 0.909 (P19-DYA) with mean PIC of 0.8105±0.01 in the present study. The average heterozygosity was observed to be 0.8347±0.01 ranging from 0.7584 (SRCRSP5) to 0.9156 P19 (DYA) across all loci.

**Conclusion:**

Microsatellite analysis revealed high level of polymorphism across studied microsatellite markers and informativeness of the markers for genetic diversity analysis studies in Gaddi goats. This high level of polymorphism can be utilized to plan future association studies to exploit the uniqueness and adaptability of indigenous Gaddi goat breed of Western Himalayas. Most studied microsatellite markers had desired neutrality, thus proving to be good candidates for genetic characterization and diversity analysis in Gaddi breed of goats also.

## Introduction

Indian goat breeds are recognized as an invaluable component of world’s goat genetic resources. Of the total goat population, 92.76% of goats are found in Asia and Africa. China, India, Pakistan and Bangladesh possess 35.36, 25.46, 10.79 and 7.05% of the total goat of Asia, respectively [1].

“Gaddi” also known as “White Himalayan goat” is the predominant goat breed of high altitude, Western temperate Himalayas with its true home tract in hills of Himachal Pradesh but distribution extending to adjoining hilly areas of Jammu and Kashmir and Uttrakhand [2]. In Himachal Pradesh, these goats are reared by traditional “Gaddi” shepherds, a distinct tribe of nomadic pastoralists leading to its nomenclature as “Gaddi” breed. These goats are highly suited for prevalent migratory production system as practiced by these pastoralists since ages. Gaddi is a medium-sized goat usually with white coat color, long horns, long drooping ears and convex nose line. The breed is amply described in terms of its geographical distribution, morphological characteristics, production attributes and cytogenetic profile [2-4] but little information [5] is so far available on molecular genetic characteristics of the breed and intra-population genetic diversity analysis.

Microsatellites have been widely used at present as standard and efficient method to characterize genetic variation among breeds and to estimate genetic diversity [6] and as to assign individuals to a breed [7,8]. Microsatellite have proved to be efficient molecular tools for diversity analysis in farm animals due to their several advantages like random distribution across the genome, high degree of ­polymorphism, co- dominance, neutrality with respect to selection and possibility of automated scoring of genotypes and have been used to analyze genetic variations in cattle, sheep, goat, pigs, buffaloes, horses and chickens [9]. But, molecular characterization studies on Gaddi goat breed using microsatellite markers are also limited [5,10]. Microsatellites, also known as simple sequence repeats or short tandem repeats, are tandemly repeating sequences of 2-6 bp of DNA, which have been demonstrated to be polymorphic in a number of eukaryotic genome [11]. Since microsatellites are polymorphic, they act as extremely useful markers for comparative study of genetic variation, parentage estimation, linkage map analysis and could well be the marker of choice for analysis of population structure in both wild [12] and domesticated [13] species.

The present study was therefore undertaken to genetically characterize Gaddi goat germplasm using suitable goat specific microsatellite markers for analyzing the prevalent genetic diversity within existing breed population, a pre-requisite for undertaking future genetic improvement and breed conservation program for the breed.

## Materials and Methods

### Ethical approval

During collection of blood samples from goats, attention had been paid to minimize pain to the animals and all the samples collection was carried out in accordance with the guidelines laid down by the International Animal Ethics Committee and prevailing local laws and regulations. The approval for carrying out this study was taken from the Institutional Animal Ethics Committee.

### Collection of blood samples

**Figure-1:**
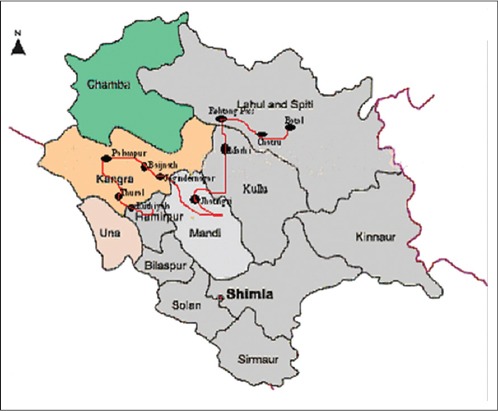
Migratory route along with distribution of Gaddi goat in Himachal Pradesh.

Totally, 75 venous blood samples were collected at random from genetically unrelated animals of Gaddi goat breed belonging to either sex or different age groups from its from different locations of the natural breeding tract (Kullu, Kangra Mandi, Bilaspur Chamba, Kinnaur, lahaul and Spiti, adjoining areas of Palampur and other districts of Himachal Pradesh as shown in Figure-1.

### Isolation of DNA samples

Of these, 51 blood samples with desired quantity up to 5-10 ml and quality ensuring unrelatedness from different individuals such that the genomic DNA isolated was of high molecular weight (>100-150 kb) and free from impurities like RNA, protein, organic solvent or salt and thus further processed for DNA isolation by phenol-chloroform extraction method [14]. The quality of DNA was assessed through 0.7% horizontal mini-submarine agarose gel electrophoresis. The purity of DNA was assessed by calculating ratio of optical densities at 260 nm and 280 nm. The samples with OD ratio (OD260/OD280) ranging from 1.7 to 1.9 was used in subsequent experiments and without any smear in 0.7% agarose gel electrophoresis.

### Primer preparation

15 FAO (DADIS MoDAD) recommended goat specific microsatellite markers selected from the list of 30 heterologous primers recommended for goat genetic diversity analysis namely ILSTS005, TGLA53, ETH10, OarFCB48, MAF70, ILSTS029, SRCRSP5, BM6444, INRABERN172, MAF065, DRBPP1, P19 (DYA), OarAE54, SPS113, TRBV6 (Table-1), which gave amplification were included in the analysis.

**Table-1:** Primer characteristics of 15 microsatellite loci amplified in Gaddi goats.

Marker	Primer sequence(5→3’)	Base pairs	Optimized annealing temperature (°C)
	
	Forward	f’	
	
	Reverse	r’	
SRCRSP5	GGA CTC TAC CAA CTG AGC TAC AAG	24	55
	TGA AAT GAA GCT AAA GCA ATG C	22	
ILSTS005	GGA AGC AAT TGA AAT CTA TAG CC	23	55
	TGT TCT GTG AGT TTG TAA GC	20	
MAF065	AAA GGC CAG AGT ATG CAA TTA GGA G	25	58
	CCA CTC CTC CTG AGA ATA TAA CAT G	25	
MAF70	CAC GGA GTC ACA AAG AGT CAG ACC	24	65
	GCA GGA CTC TAC GGG GCC TTT GC	23	
OarFCB48	GAG TTA GTA CAA GGA TGA CAA GAG GCA C	28	58
	GAC TCT AGA GGA TCG CAA AGA ACC AG	26	
OarAE54	TAC TAA AGA AAC ATG AAG CTC CCA	24	58
	GGA AAC ATT TAT TCT TAT TCC TCA GTG	27	
SPS113	CCT CCA CAC AGG CTT CTC TGA CTT	24	58
	CCT AAC TTG CTT GAG TTA TTG CCC	24	
INRABERN172	CCA CTT CCC TGT ATC CTC CT	20	58
	GGT GCT CCC ATT GTG TAG AC	20	
ILSTS029	TGT TTT GAT GGA ACA CAG	18	55
	TGG ATT TAG ACC AGG GTT GG	20	
TGLA53	GCT TTC AGA AAT AGT TTG CAT TCA	24	55
	ATC TTC ACA TGA TAT TAC AGC AGA	24	
BM6444	CTC TGG GTA CAA CAC TGA GTC C	22	65
	TAG AGA GTT TCC CTG TCC ATC C	22	
ETH10	GTT CAG GAC TGG CCC TGC TAA CA	23	55
	CCT CCA GCC CAC TTT CTC TTC TC	23	
TCRVB6	GAG TCC TCA GCA AGC AGG TC	20	55
	CCA GGA ATT GGA TCA CAC CT	20	
DRBP1	ATG GTG CAG CAG CAA GGT GAG CA	23	58
	GGG ACT CAG TCT CTC TAT CTC TTT G	25	
P19 (DYA)	AAC ACC ATC AAA CAG TAA GAG	21	55
	CAT AGT AAC AGA TCT TCC TAC A	22	

### Processing of DNA samples

The microsatellite loci were amplified in programmable thermal cycler (Bio-Rad, S 1000) after optimization. The polymerase chain reaction (PCR) program used involved initial denaturation at 94°C for 3 min and 30 cycles of denaturation at 94°C for 30 s, annealing for 45 s, extension at 72°C for 45 s and final extension at 72°C for 10 min. Documentation of PCR product was done in 1.5% agarose gel electrophoresis at 2-5 v/cm. The PCR products for different microsatellite loci were resolved on 6% denaturing (urea) polyacrylamide gels along with 50 and 100 bp DNA ladders at 40-45 w. Microsatellite alleles were visualized by silver staining.

### Statistical analysis

The microsatellite genotype data were analyzed using PopGene version (1.3.1) software to calculate allele frequencies, observed and effective number of alleles, observed and effective heterozygosities and polymorphism information content (PIC) in the population.

## Result and Discussion

Various measures of genetic diversity obtained in the present study with Gaddi goats are presented in Table-2. All 15 microsatellite loci that have been identified to be polymorphic in a variety of domestic goats [15-21] amplified successfully in Gaddi breed as well and produced definite banding patterns from which individual genotypes could be ascertained.

**Table-2:** Measures of genetic diversity at each microsatellite locus in Gaddi goats.

Microsatellite locus	Sample size	Observed number of alleles	Effective number of alleles	Allele size range (bp)	Observed heterozygosity	Expected heterozygosity	Average heterozygosity	Nei’s genetic distance (D)	Shannon’s information index	PIC
ILSTS005	102	6	4.7725	172-218	0.6471	0.7983	0.7905	0.7905	1.6297	0.758
TGLA53	102	9	8.4448	126-260	0.7843	0.8903	0.8816	0.8816	2.1613	0.8694
ETH10	102	8	6.6015	200-210	0.7451	0.8569	0.8485	0.8485	1.9447	0.8299
OarFCB48	102	7	4.3788	149-173	0.7059	0.7793	0.7716	0.7716	1.6349	0.7373
MAF70	98	7	5.8993	134-168	0.7347	0.8390	0.8305	0.8305	1.8362	0.8077
ILSTS029	102	7	5.7544	148-170	0.6471	0.8344	0.8262	0.8262	1.8143	0.803
SRCRSP5	102	5	4.1384	156-178	0.7451	0.7659	0.7584	0.7584	1.4537	0.7148
BM6444	98	10	6.1961	118-200	0.8163	0.8473	0.8386	0.8386	2.0172	0.8195
INRABERN172	100	9	5.1706	234-256	0.9200	0.8147	0.8066	0.8066	1.8430	0.7829
MAF065	100	9	6.2578	116-158	0.7200	0.8487	0.8402	0.8402	1.9813	0.821
DRBP1	102	15	10.4040	195-229	0.8039	0.9128	0.9039	0.9039	2.4969	0.8958
P19(DYA)	102	15	11.8497	160-196	0.8824	0.9247	0.9156	0.9156	2.5778	0.909
OarAE54	100	14	7.9491	115-138	0.7200	0.8830	0.8742	0.8742	2.3064	0.8263
SPS113	100	7	5.8685	134-158	0.6200	0.8380	0.8296	0.8296	1.8078	0.8061
TRBV6	98	7	5.1249	17-255	0.7347	0.8132	0.8049	0.8049	1.7503	0.7771
Mean±SE	101±0.42	9.00±0.82	6.5874±0.56		0.7484±0.02	0.8431±0.01	0.8347±0.01	0.8347±0.01	1.9504±0.08	0.8105±0.01

SE=Standard error, PIC=Polymorphism information content

A total of 135 distinct alleles were detected (Table-2) over 15 studied microsatellite loci with a mean observed number of alleles (Na) of 9.0000±0.82 alleles per locus. The allele size ranged between 115 bp (OarAE54) and 256 bp (INRABERN172). These microsatellites exhibited a high level of polymorphism as revealed by a wide range of alleles varying from 5 (SRCRSP5) to 15 (DRBP1 and P19 [DYA]). The mean effective number of alleles (Ne) was 6.5874±0.56. The mean effective number of alleles was less than the observed values across all loci and ranged from 4.1384 (SRCRSP5) to 11.8497 in P19 (DYA). The overall allelic diversity considered to be a reasonable indicator of genetic variation within the population [4,9,22] displayed high genetic variation in Gaddi goat breed. The FAO had specified at least 4 distinct alleles per locus for proficient judgment of genetic distance between breeds. Hence, all 15 microsatellite markers studied, exhibited ample polymorphism for evaluating intra-population genetic variability in Gaddi breed. The results obtained in Gaddi goats corroborated well with Kanniadu goat [23] who reported observed number of alleles between 5 (RM4) and 13 (RM088, OarE129) with mean value of 8.64±0.48 and effective number of alleles from 1.45 (ILSTS34) to 7.89 (ILSTS033 and OMHC1) with mean value of 4.22±0.34.

Heterozygosity is an appropriate measure of genetic variability within a population when populations are expanding. Therefore, heterozygosity values were used as an estimate for variability of studied goat population. The observed and expected heterozygosity values on the basis of allele frequency are also given in Table-2. The mean observed and expected heterozygosities were 0.7484±0.02 (range of 0.6200 (SPS113) to 0.9200 (INRABERN172)) and 0.8431±0.01 (range of 0.7659 (SRCRSP5) to 0.9247 P19 (DYA)) respectively. Most of the loci showed relatively higher expected heterozygosity values that might be due to low selection pressure, large population size and immigration of new genetic materials.

The PIC values, which denotes the statistical assessment of informativeness of a marker were high and ranged from 0.7148 (SRCPS5) to 0.909 (P19 [DYA]) with mean PIC of 0.8105±0.01 (Table-2). This may be due to the fact that there was increased level of heterozygosity and allele richness in the population which are the good indicators of genetic polymorphism in present study on Gaddi goats. These values are indicative of the fact that the markers used were highly informative for analysis of genetic diversity in Gaddi goat breed [24]. The genetic marker showing PIC values higher than 0.5 are normally considered as informative in population genetic analysis [25]. Mean PIC value of 0.48, lower than the values obtained in the present study, was earlier reported in three Indian goat breeds (Sirohi, Jamunapari and Barbari) using cattle microsatellite markers [26]. The present PIC values were comparable with Chinese goat breeds (0.746-0.800) as reported by [27] using ovine microsatellite markers and mean value of 0.82 in Berari goats [28]. In contrast, [16] obtained lower PIC values for Korean (0.350), Chinese (0.620) and Saanen (0.570) goats. Further [21] reported PIC estimates ranging from 0.746 to 0.800 using ovine microsatellite markers that were comparable with Chinese goat breeds, and thus consistent with findings of the present study in Gaddi goats. Similarly [29] reported PIC values from 0.271 (OarJMP29) to 0.878 (ILSTS 082) in Sangamneri goats across loci with mean of 0.711.

The genetic variability of a population is usually measured as average heterozygosity per locus while the gene differences between two populations may be measured by Nei’s standard genetic distance. The values for these measures varied from 0.7584 (SRCRSP5) to 0.9156 P19 (DYA) with mean genetic distance of 0.8347±0.01. The mean Shannon’s index value was high (1.9504±0.08) showing higher gene diversity in the existing population.

## Conclusion

In the present investigation, an attempt has been made to genetically characterize “Gaddi” goat population in its breeding tract in HP using 15 FAO recommended goat specific microsatellite markers. Microsatellite analysis revealed high level of polymorphism and informativeness of studied microsatellite markers in genetic diversity analysis in Gaddi goats. The high PIC values as observed in the study are indicative of high informativeness of studied markers for genetic diversity analysis in Gaddi goat breed. Most studied microsatellite markers had desired neutrality, thus proving to be good candidates for genetic characterization and diversity analysis in Gaddi breed of goats also. The information gathered could be utilized to plan breeding, improvement and conservation programs for this valuable goat germplasm resource and future association studies to exploit its unique adaptability traits. The significant level of variability in this population reflects that the Gaddi goat population contains a valuable and substantial amount of genetic diversity among the studied breed and thus there is good scope for bringing effective genetic improvement, conservation and designing future breeding policies for these goats.

## Authors’ Contributions

GS and YT planned and designed the study. GS and AK collected the samples, GS and VS performed test and analyzed the data. All authors participated in draft and revision of the manuscript. All authors read and approved the final manuscript.
